# HnRNP-F promotes the proliferation of bladder cancer cells mediated by PI3K/AKT/FOXO1

**DOI:** 10.7150/jca.50490

**Published:** 2021-01-01

**Authors:** Fei Li, Weiwei Xie, Yunze Fang, Kunfeng Xie, Wendong Liu, Lina Hou, Wanlong Tan

**Affiliations:** 1 Department of Urology, Nanfang Hospital, Southern Medical University, Guangzhou, Guangdong 510515, P.R.China.; 2 Department of Healthy Management, Nanfang Hospital, Southern Medical University, Guangzhou, Guangdong 510515, P. R. China.

**Keywords:** Heterogeneous nuclear ribonucleoprotein F, Phosphoinositide 3‑kinase/protein kinase B signalling pathway, Bladder cancer, Forkhead box O1, Proliferation

## Abstract

Our previous study showed that heterogeneous nuclear ribonucleoprotein F (hnRNP-F) could induce epithelial-mesenchymal transition and metastasis in bladder cancer (BC), however, the role and mechanism of hnRNP-F in mediating the proliferative ability of BC cells remain unclear. HnRNP-F promoted the proliferation of BC cells by using BC cell lines and cell counting kit-8 (CCK8), colony formation and flow cytometry assays *in vitro*. Furthermore, the association of hnRNP-F with the phosphoinositide 3‑kinase (PI3K)/protein kinase B (AKT) signalling pathway was confirmed by western blotting after bioinformatic analysis. HnRNP-F expression was significantly decreased by treatment with the PI3K/AKT signalling pathway inhibitor LY294002, whereas hnRNP-F knockdown did not significantly affect PI3K or AKT expression, suggesting that hnRNP-F is likely a downstream target of the PI3K/AKT pathway. Forkhead box O1 (FOXO1) is a molecule downstream of PI3K/AKT and can be inhibited by phosphorylation. In addition, chromatin immunoprecipitation (ChIP) and luciferase reporter assays indicated that FOXO1 expression was negatively correlated with hnRNP-F expression as FOXO1 was found to bind to the promoter region of hnRNP-F mRNA and inhibit its transcription. To sum up, our findings suggest that hnRNP-F expression is regulated by the PI3K/AKT-mediated phosphorylation of FOXO1, with phosphorylation inhibiting FOXO1, which subsequently allows hnRNP-F to promote proliferation. This finding is a novel discovery in BC and could help reveal the mechanism of BC progression.

## Introduction

Bladder cancer is one of the most common malignancies in adults, with high morbidity and mortality rates worldwide 1. Multi-gene, multi-factor and multi-step processes, including aberrant cell proliferation, are involved in the carcinogenesis and progression of BC 2. Increased BC cell proliferation can contribute substantially to carcinogenesis. The pathological mechanism of BC cell proliferation is a complex process involving the synthesis and degradation of molecules and the activation and deactivation of signalling pathways 3. The signalling pathways mediating cell proliferation are important and complex, therefore, identifying a molecule reliably and related mechanism involved in the aberrant proliferation of BC cells may be helpful for treatment and better prognosis.

The pathological proliferation of cancer cells involves a large number of genes and proteins and multiple signalling pathways, such as the p21, MAPK, and phosphoinositide 3‑kinase (PI3K)/protein kinase B (AKT) signalling pathways 3, 4. Activated PI3K phosphorylates and activates AKT, which plays key roles in various cancers in an extensive range of cellular regulatory processes, such as cell proliferation, invasion, migration and metabolism 5, 6. AKT can phosphorylate various substrates, which can have inhibitory or stimulatory effects; thus, AKT can exert different downstream effects depending on the target proteins affected 7-9. Forkhead box O1 (FOXO1) is a molecule downstream of PI3K/AKT and can be inhibited by phosphorylation 8, which subsequently results in suppression of apoptosis and regulation of the cell cycle in prostate cells 10. Recent studies have demonstrated that FOXO1 expression is decreased in BC tissue compared with non-cancerous bladder mucosal tissue and that FOXO1 is associated with a good prognosis and considered a tumour suppressor in BC 11-13.

Heterogeneous ribonucleoprotein F (hnRNP-F) belongs to the hnRNP family, which is involved in multiples aspects of translational regulation, including pre- and mature mRNA transcription, mRNA stabilization, posttranscriptional modificaiton and alternative splicing 14. HnRNP-F was demonstrated to play an oncogenic role in many cancers including BC 14. In our previous study, hnRNP-F was shown to mediate the stabilization of Snail1 mRNA by binding to its 3' untranslated region (3'UTR), subsequently regulating epithelial-mesenchymal transition (EMT) in BC 15. However, the role of hnRNP-F in BC cell proliferation remains poorly understood.

To explore the role and mechanism of hnRNP-F in mediating the proliferative ability of BC cells, in this study, we first show that hnRNP-F could promote the proliferation of cells in BC EJ and UMUC3 cells. Furthermore, Kyoto Encyclopedia of Genes and Genomes (KEGG) pathway enrichment analysis based on 403 BC dataset from The Cancer Genome Atlas (TCGA) indicated that the PI3K/AKT pathway is significantly enriched in BC tumour tissue samples with up-regulated hnRNP-F expression. The further results showed that hnRNP-F is likely a downstream target of the PI3K/AKT pathway, and FOXO1 could bind to the promoter region of hnRNP-F mRNA and inhibit its transcription. Our findings provide novel insights into the function and mechanisms of PI3K/AKT/FOXO1-mediated regulation of hnRNP-F in BC pathogenesis, indicating that hnRNP-F could affect BC proliferation and therefore cancer progression.

## Materials and methods

### Antibodies

A rabbit polyclonal antibody against hnRNP-F was acquired from Novus Biologicals (Colorado, USA), and rabbit monoclonal antibodies against AKT, p-AKT, PI3K, p-PI3K, FOXO1, and p-FOXO1 were acquired from Cell Signaling Technology (CST) (Massachusetts, USA). An IgG rabbit polyclonal antibody, IgG mouse polyclonal antibody, Alexa Fluor 594-conjugated goat anti-rabbit IgG (H+L) and Alexa Fluor 488-conjugated goat anti-mouse IgG (H+L) were purchased from Proteintech (Guangzou, China). HRP-conjugated goat anti-rabbit IgG (H+L) and HRP-conjugated goat anti-mouse IgG (H+L) antibodies and antibodies against glyceraldehyde 3-phosphate dehydrogenase (GAPDH) were also purchased from Proteintech (Guangzou, China).

### Cell culture

The human BC cell lines 5637, T24, EJ and UMUC3 were cultured in RPMI-1640 medium (Gibco, California, USA) supplemented with 10% foetal bovine serum (Serana, Berlin, Germany) and 1% penicillin/streptomycin at 37 °C in a humidified incubator in a 5% CO_2_ atmosphere. The cell lines were authenticated by short tandem repeat (STR) profiling upon receipt and were propagated for <6 months after resuscitation. The PI3K/AKT signal pathway inhibitor LY294002 was purchased from Procell (Wuhan, China) and was used according to the instruction.

### Western blotting

Protein lysates derived from BC cells were separated by 12% SDS-PAGE gels and then transferred to 0.45-μm PVDF membranes. After being blocked with 10% skim milk for 30 minutes at room temperature, the membranes were incubated with antibodies against hnRNP-F, AKT, p-AKT, PI3K, p-PI3K, FOXO1, p-FOXO1 (all 1:1000) and GAPDH (1:5000) at 4 °C overnight, followed by an incubation with appropriate HRP-conjugated secondary antibodies (1:5000). Immunoreactivity was visualized by ECL (Fdbio Science, Hangzhou, China) and an enhanced chemiluminescence (ECL, Pierce, Rockford, IL, USA) detection system for blot visualization. The protein levels were normalized to those of GAPDH.

### Real-time quantitative polymerase chain reaction (RT-qPCR)

RT-qPCR was performed according to the instructions of a standard SYBR Green PCR kit (Takara, Japan) on an Applied Biosystems 7500 Real-Time PCR system (Foster City, CA). GAPDH was used for normalization. The primer sequences used in our study are shown in Table S1.

### Stable knockdown of hnRNP-F expression

To stably suppress the expression of hnRNP-F in the EJ and UMUC3 cell lines, a lentivirus encoding a short hairpin RNA (shRNA) targeting hnRNP-F (sh-hnRNP-F) with the sequence 5'-GGUACAUUGAGGUGUUCAATT-3' was constructed (GenePharma, Suzhou, China). After transduction with the sh-hnRNP-F virus or a negative control virus, cells were screened using 5 µg/ml puromycin (Sigma, Missouri, USA). The transfection efficiency was assessed by western blotting performed as described in subsection 2.3.

### Cell counting kit-8 (CCK8) assay

The proliferation of EJ and UMUC3 cells was evaluated by using CCK8 (Dojindo, Japan) according to the manufacturer's instructions. In brief, 10 μl of CCK8 solution was added to the culture medium and incubated for 2 h in a 5% CO_2_ atmosphere at 37 °C. The absorbance was measured at a wavelength of 450 nm, with a reference wavelength of 570 nm. The cell proliferation assay was performed on days 1, 2, 3, 4, 5 and 6.

### Colony formation assay

EJ and UMUC3 cells (600 cells per well) were added to 6-well plates and cultured for 14 days. The resultant colonies were washed with PBS, fixed with 4% paraformaldehyde for 30 min, and stained with Giemsa (Solarbio, Beijing, China). The colonies were photographed with an inverted microscope (Axioskop 2 Plus; Zeiss, Germany) and counted.

### Flow cytometry assay

The cell cycles of EJ and UMUC3 cells were examined by flow cytometry. Cells in log-phase growth were harvested and fixed with 70% ice-cold alcohol overnight. The cell pellets were treated with 100 µg/ml RNase, stained with 50 µg/ml propidium iodide (PI) and incubated for 30 min, after which time they were analysed by flow cytometry (FACSCalibur, Becton Dickinson).

### Chromatin immunoprecipitation (ChIP) assays

EJ and UMUC3 cells transfected with pENTER or pENTER-hnRNP-F were collected, and ChIP assays were performed with the ChIP-IT High Sensitivity® Kit (50340, Active Motif North America) according to the manufacturer's instructions. In brief, BC cells were fixed and sonicated to shear DNA into fragments of 200~1000 bp, and the following immunoprecipitation antibodies were then added: anti-FOXO1 (CST) and rabbit IgG (sc-2027, Santa Cruz, California, USA). Specific primers (Table S1) were used for further analysis by conventional PCR and qPCR as described previously 15. One-fiftieth of the starting amount of chromatin (input) was used as the control.

### Luciferase reporter assay

A dual-luciferase reporter assay was used to investigate the relationship between FOXO1 and hnRNP-F. In brief, the firefly luciferase reporter vector pENTE-hnRNP-F and a Renilla luciferase plasmid (pRL-TK) were transiently co-transfected into EJ and UMUC3 cells pretreated with FOXO1-specific or control shRNA. PRL-TK was used to normalize for transfection efficiency. After transfection activity, luciferase activity was measured by using a dual-luciferase reporter assay kit (E1960, Promega, Wisconsin, USA) according to a previously reported protocol 15.

### Data acquisition and preprocessing

The RNA-seq raw count expression profile and fragments per kilobase million (FPKM)-normalized expression profile of 403 samples from the BC (BLCA) dataset were obtained from The Cancer Genome Atlas (TCGA) programme data portal (https://cancergen-ome.nih.gov/). The data were used for KEGG enrichment analysis (https://www.kegg.jp/kegg/pathway). Furthermore, the promoter sequence of hnRNP-F was identified by using ALGGEN in PubMed database.

### Statistical analysis

Data are presented as the mean±standard deviation (SD), and all experiments were repeated three times. Student's t-test was used to analyse continuous data. All statistical analyses were performed with SPSS 20.0 software (SPSS, Inc., Chicago, USA). *P* <0.05 was considered significant.

## Results

### HnRNP-F expression is up-regulated in human BC

**Figure 1:**
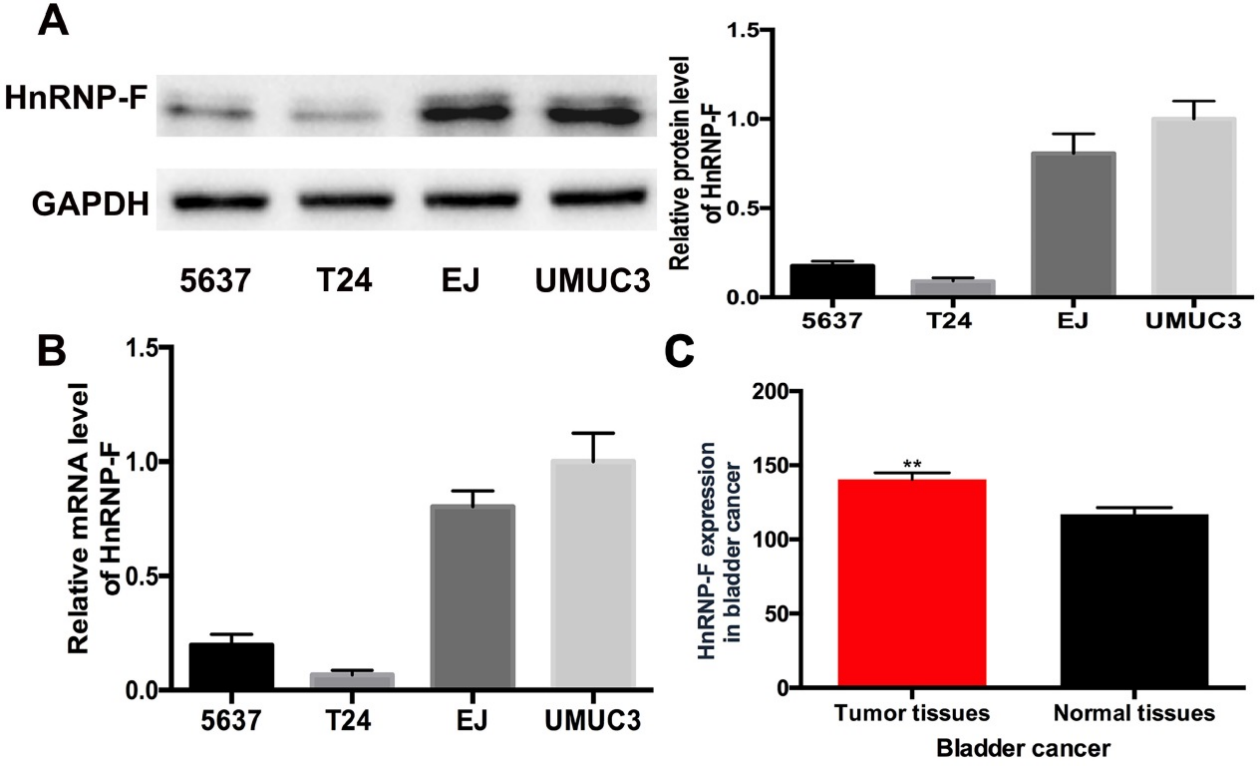
** HnRNP-F expression was up-regulated in BC. A-B.** HnRNP-F protein (A) and mRNA (B) were detected in the 5637, T24, EJ and UMUC3 cell lines by western blot and RT-qPCR. **C.** HnRNP-F mRNA expression in BC and normal tissue samples was evaluated by TCGA database analysis (** *P*<0.01).

The expression levels of hnRNP-F in human BC cell lines were quantified by western blot and RT-qPCR assays. HnRNP-F protein level was significantly increased in EJ and UMUC3 cells compared with 5637 and T24 cells (*P*<0.01, Figure 1A), which was consistent with the results for hnRNP-F mRNA levels (*P*<0.01, Figure 1B). Given the high expression of hnRNP-F in EJ and UMUC3 cells, therefore, EJ and UMUC3 cells were used for further analysis. Furthermore, the analysis of hnRNP-F mRNA expression using the TCGA database showed that hnRNP-F transcription was significantly increased in BC tumour tissue compared with non-cancerous tissue (Figure 1C). Briefly, hnRNP-F is up-regulated in human BC EJ and UMUC3 cells.

### HnRNP-F is involved in promoting BC cell proliferation

**Figure 2:**
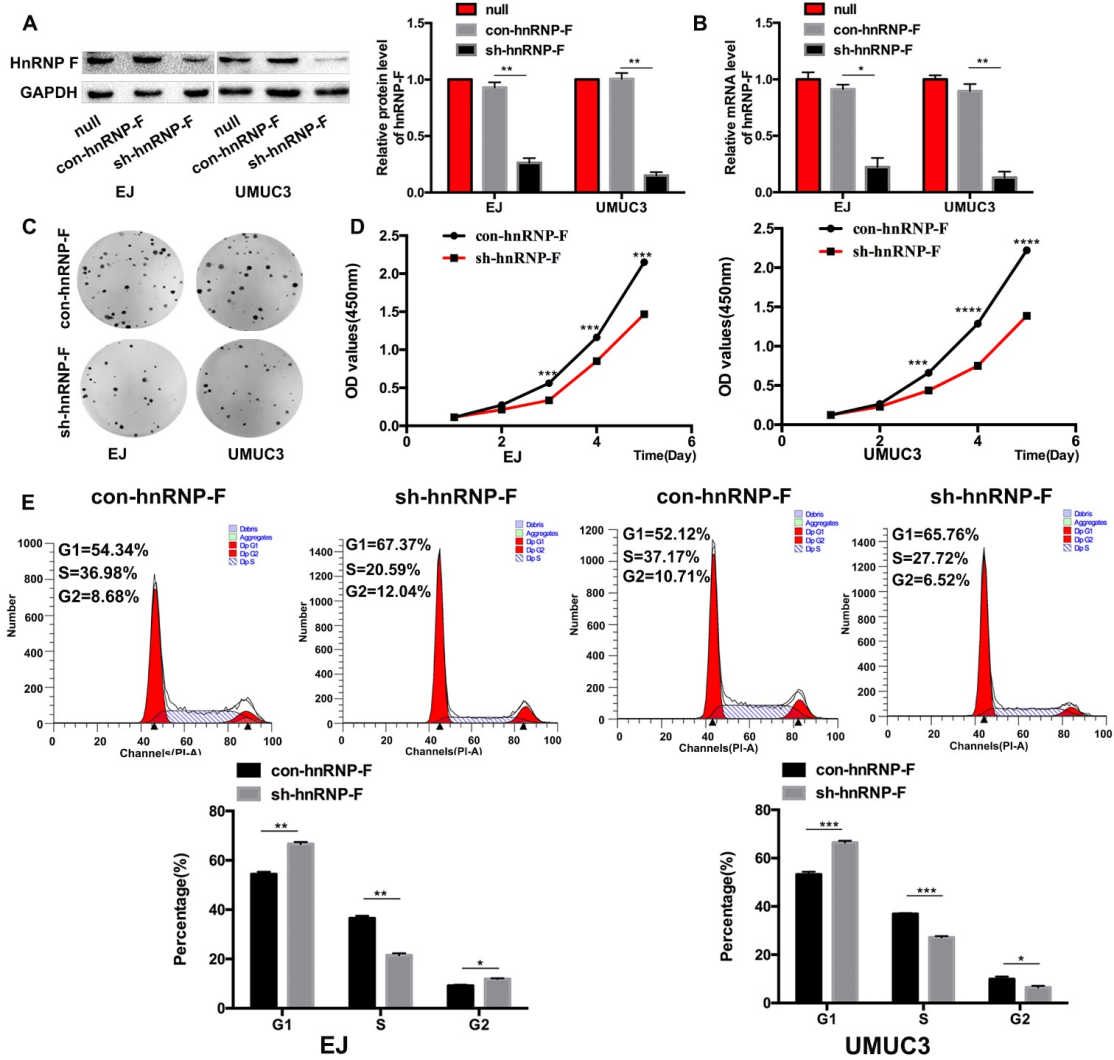
Knockdown of hnRNP-F expression inhibited cell proliferation and cell cycle progression in BC cells *in vitro* (* *P*<0.05, ** *P*<0.01 and *** *P*<0.001). A-B. Transfection of sh-hnRNP-F was performed to establish EJ and UMUC-3 cell lines with stable knockdown of hnRNP-F expression. The hnRNP-F levels in EJ and UMUC3 cells after hnRNP-F knockdown were measured by western blotting (A) and RT-qPCR (B). **C-D.** Colony formation (C) and CCK8 (D) assays were performed to evaluate the effect of hnRNP-F knockdown on the proliferation of EJ and UMUC3 cells. **E.** The cell cycle distributions of EJ and UMUC3 cells were analysed by flow cytometry. In the bar graphs, the data are shown as the mean ± SD.

To explore the effect of hnRNP-F on the proliferation of human BC cells, EJ and UMUC3 cells were treated with sh-hnRNP-F to suppress endogenous hnRNP-F expression and evaluated. Western blot and RT-qPCR analyses showed that hnRNP-F expression was significantly downregulated in sh-hnRNP-F-transduced cells compared with control cells transduced with a negative control virus (con-hnRNP-F cells; EJ, *P*<0.05 and UMUC3, *P*<0.01; Figure 2A and B).

Then, the growth and proliferation of the transduced cells were compared with those of control cells to assess the functions of hnRNP-F. The colony formation capacity of hnRNP-F-depleted cells was significantly suppressed compared with that of control cells (EJ, *P*<0.01 and UMUC3, *P* <0.001; Figure 2C). Furthermore, in a CCK8 cell proliferation assay, the *in vitro* growth of hnRNP-F-depleted EJ and UMUC3 BC cells was significantly inhibited at day 3 (EJ, *P*<0.001 and UMUC3, *P*<0.0001; Figure 2D).

In addition, the cell cycle distribution was evaluated by flow cytometry, and the results showed a significantly increased percentage of G1-phase cells among both EJ and UMUC-3 cells with hnRNP-F knockdown compared with the corresponding con-hnRNP-F-transduced cells (*P*<0.01). In contrast, the percentage of S-phase cells was significantly decreased in the knockdown cells (*P*<0.01) (Figure 2E). These findings suggest that hnRNP-F promotes proliferation in BC cells.

### HnRNP-F is a likely target of the PI3K/AKT pathway

**Figure 3:**
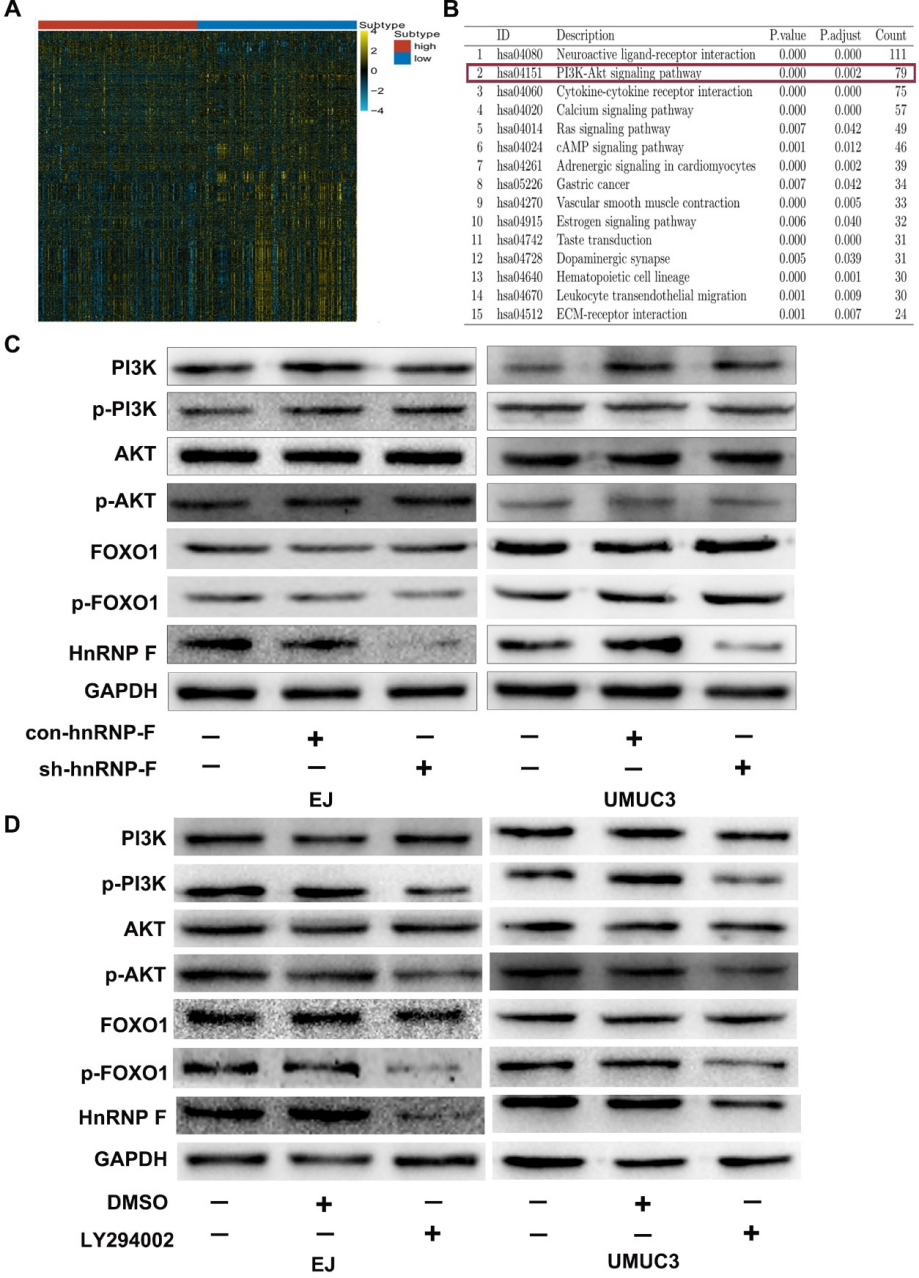
HnRNP-F is a likely target of the PI3K/AKT pathway (* *P*<0.05, ** *P*<0.01 and *** *P*<0.001). A. The differentially expressed genes between two groups of patients in the TCGA database stratified by hnRNP-F expression were identified by enrichment analysis. **B.** The signalling pathways associated with hnRNP-F were identified by KEGG enrichment analyses. **C.** The protein levels of PI3K, p-PI3K, AKT, p-AKT, FOXO1 and p-FOXO1 in EJ and UMUC3 cells with or without hnRNP-F knockdown were measured by western blotting. **D.** The protein levels of PI3K, p-PI3K, AKT, p-AKT, FOXO1, p-FOXO1 and hnRNP-F in EJ and UMUC3 cells treated with or without LY294002 were measured by western blotting.

To further investigate the mechanism of hnRNP-F promotes proliferation in BC cells, gene enrichment analysis was performed based on the sequencing data for the 403 TCGA BC tumour tissue samples. A total of 2878 differentially expressed genes whose levels exhibited at least a 2-fold change were identified between the hnRNP-F-upregulated group and the hnRNP-F-downregulated group stratified by the mean level of hnRNP-F mRNA of TCGA BC tumours (Figure 3A). KEGG pathway enrichment analysis of the differentially expressed genes showed that many signalling pathways, such as the neuroactive ligand-receptor interaction, PI3K/AKT, calcium, Ras, and cAMP pathways, were enriched in the hnRNP-F up-regulated group (Figure 3B). Although the PI3K/AKT pathway is strongly correlated with cell proliferation in cancer 16, the association of hnRNP-F with the PI3K/AKT signalling pathway in BC has not been reported.

To investigate the relationship between hnRNP-F and the PI3K/AKT signalling pathway in BC, no changes in the protein levels of PI3K, p-PI3K, AKT and p-AKT were observed in response to knockdown of hnRNP-F expression in the EJ and UMUC3 cell lines (*P*>0.05, Figure 3C). However, the protein levels of p-PI3K, p-AKT and hnRNP-F were decreased when BC cells were treated with the PI3K/AKT signalling pathway inhibitor LY294002 (*P*<0.01, Figure 3D). Notably, the PI3K and AKT protein levels did not differ. Thus, changes in the hnRNP-F level did not affect PI3K/AKT pathway molecules, but inhibition of the activity of the PI3K/AKT pathway did decrease hnRNP-F expression.

In summary, the abovementioned results indicated that hnRNP-F is a likely target of PI3K/AKT signalling.

### FOXO1 inhibits hnRNP-F transcription

**Figure 4:**
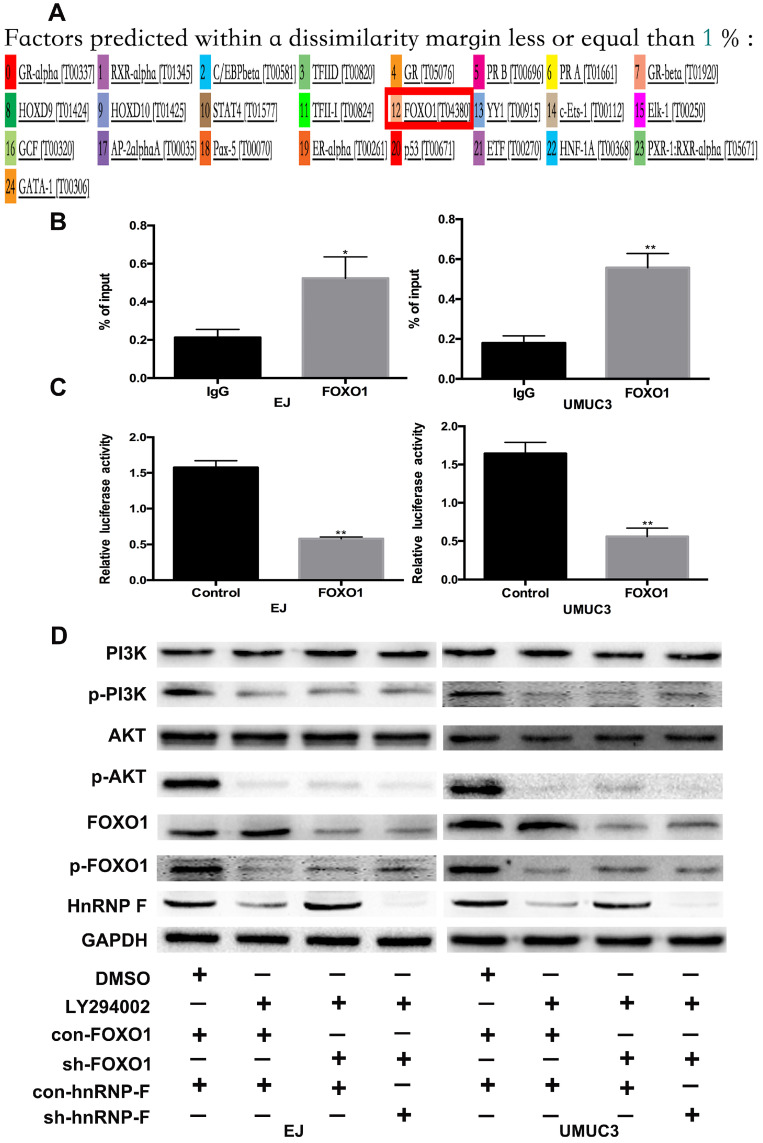
FOXO1 regulates hnRNP-F transcription (* *P*<0.05 and ** *P*<0.01). A. ALGGEN PROMO was used to predict the potential transcription factors of hnRNP-F. **B-C.** A ChIP assay (B) and a dual-luciferase reporter assay with shRNA co-transfection (C) were conducted to reveal the relationship between hnRNP-F and FOXO1. **D.** After treatment with LY294002, the protein levels of PI3K, p-PI3K, AKT, p-AKT, FOXO1, p-FOXO1 and hnRNP-F in EJ and UMUC3 cells with or without FOXO1 or hnRNP-F knockdown were measured by western blotting.

PI3K/AKT can phosphorylate downstream proteins but does not inhibit the expression of these targets 16-18. Therefore, we hypothesized that hnRNP-F could not be directly downstream of PI3K/AKT. The promoter sequence of hnRNP-F identified by a PubMed database search was used to evaluate transcription factor binding and activity on hnRNP-F via ALGGEN, and the downstream molecule FOXO1, which is regulated by PI3K/AKT, was selected to examine the association of hnRNP-F with the PI3K/AKT signalling pathway in BC (Figure 4A).

When BC cells were treated with the inhibitor LY294002, the protein level of p-FOXO1 was decreased (EJ, *P*<0.01 and UMUC3, *P*<0.05; Figure 3D), while no significant change was observed in the protein level of total FOXO1, indicating that the amount of active FOXO1 was increased. ChIP assays validated the occupancy of FOXO1 in the promoter region of hnRNP-F (EJ, *P*<0.05 and UMUC3, *P*<0.01; Figure 4B). The results of a dual-luciferase report assay with co-transfection of a FOXO1-specific shRNA performed to assess hnRNP-F promoter (nucleotide positions -1495~+ 167) activity clearly showed that hnRNP-F transcription was significantly downregulated by FOXO1 (EJ: *P*<0.01; UMUC3: *P*<0.01, Figure 4C). In brief, these findings suggest that hnRNP-F is the direct downstream target of FOXO1 (Figure 6).

### HnRNP-F is regulated by the PI3K/AKT-mediated phosphorylation of FOXO1

To further confirm whether hnRNP-F expression is regulated by the PI3K/AKT pathway through FOXO1, we first used western blotting to examine the relationship between FOXO1 and hnRNP-F under treatment with LY294002. Increased expression of hnRNP-F was detected in LY294002-treated EJ or UMUC3 cells co-transfected with sh-FOXO1+con-hnRNP-F compared with the corresponding cells co-transfected with con-FOXO1+ con-hnRNP (*P*<0.01, Figure 4D), indicating that knockdown of FOXO1 expression rescued the decrease in hnRNP-F expression induced by LY294002. However, the levels of p-PI3K, PI3K, p-AKT and AKT in EJ and UMUC3 cells did not change significantly upon FOXO1 knockdown (Figure 4D). These data suggest that the hnRNP-F protein level is regulated by the PI3K/AKT-mediated phosphorylation of FOXO1.

**Figure 5:**
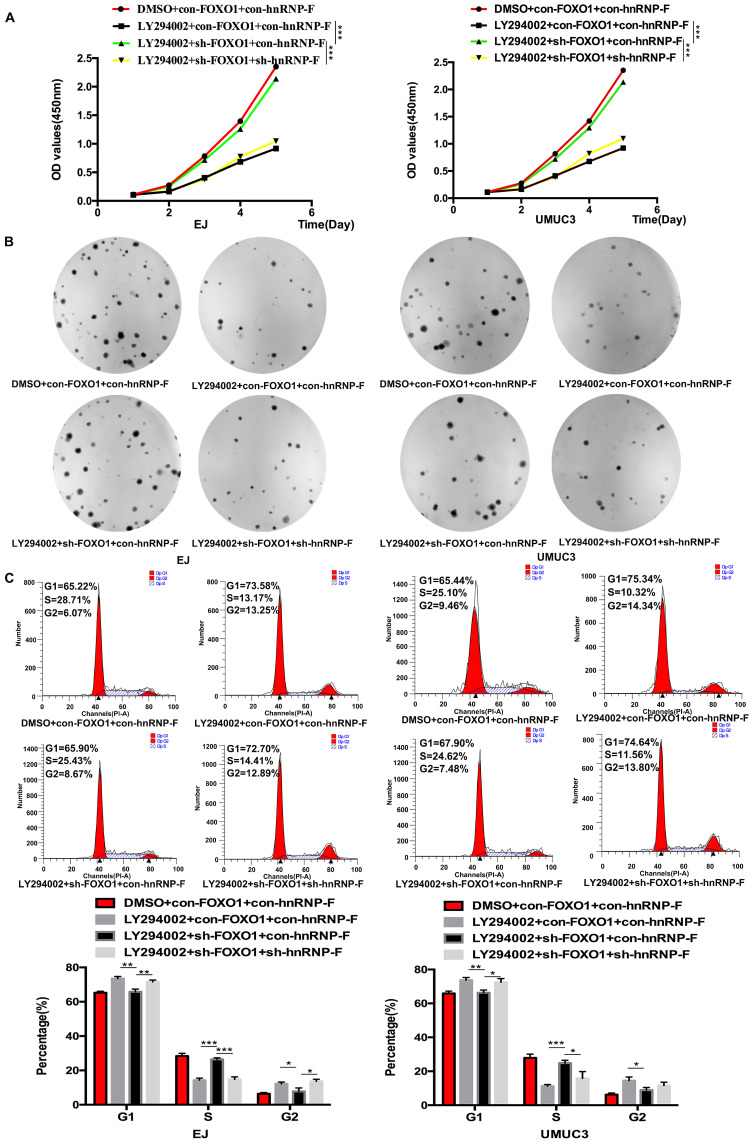
Knockdown of FOXO1 expression ameliorated the alterations in cell proliferation and the cell cycle distribution induced by LY294002 (*P<0.05, ** *P*<0.01 and *** *P*<0.001). A-B. CCK8 (A) and colony formation assays (B) were performed to determine the role of FOXO1 as an intermediary between hnRNP-F and PI3K/AKT in the proliferation of EJ and UMUC3 cells. **C.** A flow cytometry assay was conducted to examine the role of FOXO1 as an intermediary between hnRNP-F and PI3K/AKT in the cell cycle distribution of EJ and UMUC3 cells.

**Figure 6:**
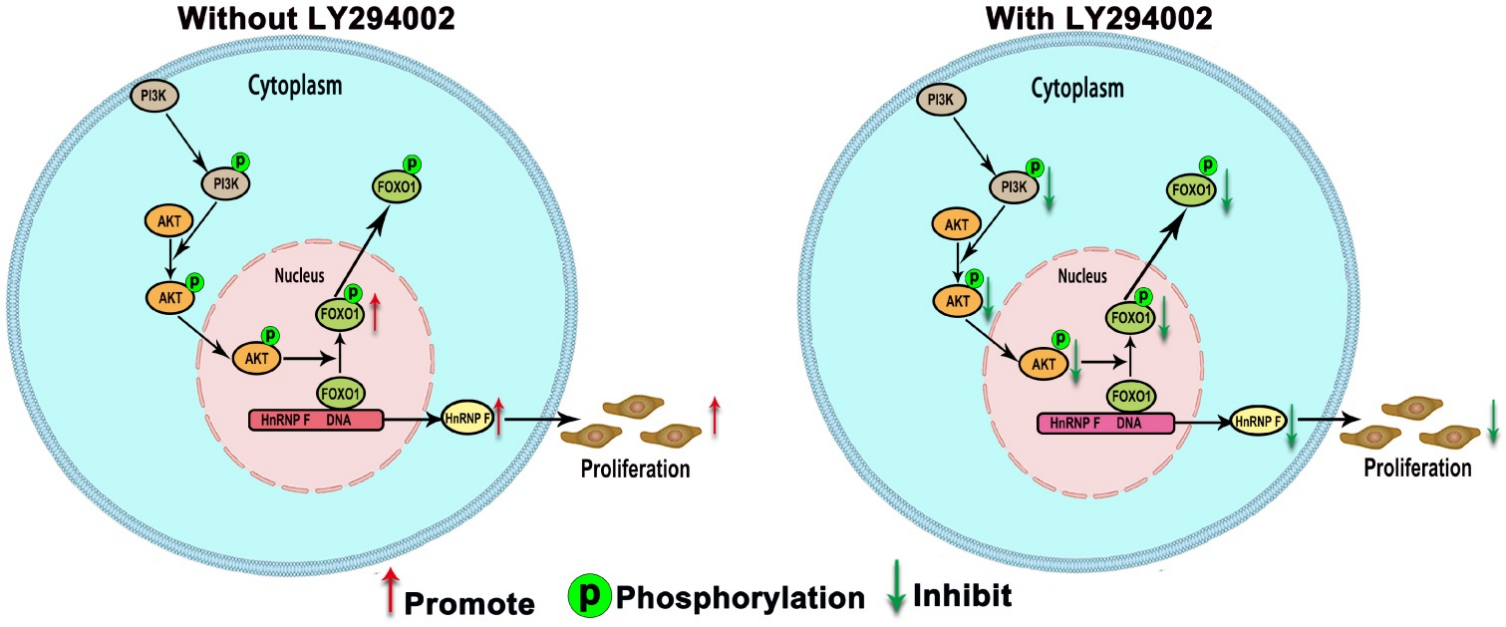
** A model illustrating our findings on the regulation of hnRNP-F by PI3K/AKT/FOXO1 is shown.** HnRNP-F expression is regulated by the PI3K/AKT-mediated phosphorylation of FOXO1, with phosphorylation inhibiting FOXO1, which subsequently allows hnRNP-F to promote proliferation.

To investigate the relationship between FOXO1 and hnRNP-F, the roles of FOXO1 in the proliferation and cell cycle progression of BC cells mediated by hnRNP-F and affected by PI3K/AKT were investigated by CCK8, colony formation and flow cytometry assays. Upon treatment with LY294002, the proliferation of sh-FOXO1+con-hnRNP-F cells was significantly more vigorous than that of con-FOXO1+con-hnRNP-F cells in both the EJ (*P*<0.001) and UMUC3 (*P*<0.001) cell lines. In contrast, the proliferation of sh-FOXO1+sh-hnRNP-F cells was significantly lower than that of sh-FOXO1+con-hnRNP-F cells in both the EJ (*P*<0.001) and UMUC3 (*P*<0.001) cell lines (Figure 5A). Furthermore, the results of the colony formation assays were consistent with those of the CCK8 assays (Figure 5B). In addition, the flow cytometry results indicated a significant increase in the G1-phase population and significant decrease in the S-phase population in EJ or UMUC3 cells transfected with sh-FOXO1 compared with the corresponding con-FOXO1 and con-hnRNP-F-co-transfected cells However, an increased G1-phase cell population and a decreased S-phase cell population were detected in EJ and UMUC3 cells after the combined knockdown of FOXO1 and hnRNP-F (Figure 5C). In summary, FOXO1 is required for the promotive effects of PI3K/AKT on cell proliferation and cell cycle progression and functions by regulating hnRNP-F in BC (Figure 6).

## Discussion

In this study, to explore the role and potential mechanism of hnRNP-F in affecting the proliferative ability of BC cells, firstly, hnRNP-F was found to be up-regulated in human BC EJ and UMUC3 cell lines by western blot and RT-qPCR assays. The knockdown of hnRNP-F could inhibit proliferation and delay cell cycle progression in EJ and UMUC-3 cells. Mechanistically, hnRNP-F expression was significantly decreased by treatment with the PI3K/AKT signalling pathway inhibitor LY294002, whereas hnRNP-F knockdown did not significantly affect PI3K or AKT expression, suggesting that hnRNP-F is likely a downstream target of the PI3K/AKT pathway. Furthermore, the ChIP and luciferase reporter assays indicated that FOXO1 expression was negatively correlated with hnRNP-F expression as FOXO1 was found to bind to the promoter region of hnRNP-F mRNA and inhibit its transcription.

BC is the 10^th^ most common form of cancer worldwide, with an estimated 549,393 new cases and 199, 922 deaths occurring globally in 2018 19. BC remains a major threat to public health because it is characterized by high recurrent rates and poor prognosis once tumors invade deeper layers 20. Aberrant proliferation is an important part of cancer development and progression and has recently received much attention 21. Therefore, it is of great importance to study the carcinogenesis and development of BC to identify effective therapeutic targets to improve prognosis.

HnRNP-F expression was reported to be increased in BC tissue, and increased hnRNP-F expression was associated with a poor prognosis in BC patients in our previous studies 15, 22. HnRNP-F is an RNA-binding protein belonging to the hnRNP family that has been implicated in multiple steps of RNA metabolism and gene expression regulation, and its involvement in cancer occurrence and progression is attracting increasing attention 14, 23. Other members of the hnRNP family have been found to affect tumour cell proliferation. In MDA-MB-231 breast cancer cells, proliferation was decreased when hnRNP-A2 and hnRNP-B1 expression was knocked down 24. Furthermore, hnRNP-L was found to have a promotive effect on the proliferation of prostate cancer cells 25. However, the role and mechanism of hnRNP-F in the proliferation of BC cells remain unclear.

HnRNP-F can affect cell proliferation in cervical cancer by modulating telomerase function through interactions with hTERC and the telomerase holoenzyme 26. In addition, our previous study showed that hnRNP-F promoted the invasion and metastasis of BC cells *in vitro* and *in vivo* 15. In this study, the proliferation of EJ and UMUC3 cells *in vitro* was inhibited when hnRNP-F was depleted. This effect of hnRNP-F depletion on cell proliferation prompted us to further explore the role of hnRNPF in cell cycle progression. Our flow cytometry results showed that in EJ and UMUC3 cells, the percentages of G1-phase cells were increased and the percentages of S-phase cells were decreased when hnRNP-F expression was knocked down, indicating that hnRNP-F promotes the G1/S phase transition in BC cells.

PI3K/AKT signalling has been shown to play pivotal roles in the proliferation and invasion of cancer cells 27. The PI3K/AKT pathway is a critical signal transduction pathway that regulates multiple cellular functions including proliferation, and the importance of PI3K/AKT signalling in BC progression has been reported 28, 29. In addition, activation of PI3K/AKT signalling has been shown to be correlated with tumour progression and clinical survival in BC patients 30. The hsa-miR-139-5p/HNRNPF axis as a novel regulatory mechanism could be associated with the modulation of major thyroid cancer signaling pathways and tumor virulence involved in RTK/RAS/MAPK and PI3K/AKT/MTOR signaling cascades 31. However, the association of hnRNP-F with PI3K/AKT signalling in BC progression remains unclear.

Aberrant activation of the PI3K/AKT pathway has been identified in a wide range of cancers 32. In BC, up to 40% of tumours exhibit constitutive activation of the PI3K/AKT pathway 33. The complex regulation of this pathway, together with the multiple mechanisms by which it can be activated, make targeting this pathway highly challenging 31. In this study, hnRNP-F was found by KEGG pathway analysis to be strongly interrelated with the PI3K/AKT pathway. Furthermore, hnRNP-F expression positively correlated with AKT activation (p-AKT), but the levels of p-AKT did not change significantly when hnRNP-F expression was changed, which indicates that hnRNP-F is the downstream of PI3K/AKT pathway. Constitutive activation of the PI3K/AKT pathway may result in high expression of hnRNP-F, as hnRNP-F is a likely target of PI3K/AKT signalling.

FOXO1 is a molecule downstream of PI3K/AKT that has been shown act as a tumour suppressor in BC 9. To date, the relationship between hnRNP-F and FOXO1 has not been reported. We investigated the association between these two proteins and found that the transcription of hnRNP-F was significantly inhibited by the binding of FOXO1 to its promoter region (nucleotide positions -1495~+ 167), as indicated by the dual-luciferase reporter assay with shRNA co-transfection. Furthermore, increased hnRNP-F expression was detected in BC cells with FOXO1 knockdown and LY294002 treatment compared with the controls. Unsurprisingly, knockdown of FOXO1 expression alone rescued the cell cycle delay and the proliferation arrest induced in EJ and UMUC3 cells by treatment with LY294002. Therefore, the above findings suggest that hnRNP-F promotes cell cycle progression and cell growth in BC and that hnRNP-F is a downstream molecule of PI3K/AKT signalling that can be regulated by the PI3K/AKT-mediated phosphorylation of FOXO1.

The current study was subject to several limitations that need to be considered when interpreting our findings. First, our findings is based on the experiments conducted in human BC EJ and UMUC-3 cells and needs to be further confirmed by animal experiments *in vivo*. Second, the genetic polymorphism factors were not considered in this study. We could not rule out that the possibility that could affect the association.

In summary, our findings demonstrate that hnRNP-F promotes the proliferation of BC cells. Furthermore, FOXO1 expression is negatively correlated with hnRNP-F expression as FOXO1 binds to the promoter region of hnRNP-F to inhibit its transcription. Mechanistically, hnRNP-F transcription is regulated by the PI3K/AKT-mediated phosphorylation of FOXO1, which is a novel discovery in BC and could be helpful for revealing the mechanism of BC progression.

## Supplementary Material

Supplementary table S1.
