# Supplementary material for: HnRNP-F promotes the proliferation of bladder cancer cells mediated by PI3K/AKT/FOXO1
Source: J Cancer. 2021 Jan 1;12(1):281–91. doi: 10.7150/jca.50490 (PMC7738822; doi:10.7150/jca.50490)
Supplement: Supplementary file 1 — Supplementary table S1. [file jcav12p0281s1.pdf]

Table S1

Gene	Upstream sequences（5'-3'）	Downstream sequences（5'-3'）
GAPDH	ACAGTCAGCCGCATCTTCTT	GACAAGCTTCCCGTTCTCAG
HnRNP F	GAAGGCTCTAGGGAAACACAAG	CACGGACATGAACTTCAGAGG
Chip HnRNP F	CCTGGTCCTGCTCTGTTGAG	TACCGGTGTCCCATGCTTTC
